# Prevalence and correlates of violence among sexual and injecting partners of people who inject drugs living with HIV in Kenya: a cross-sectional study

**DOI:** 10.1186/s12954-023-00895-7

**Published:** 2023-11-02

**Authors:** Sai Win Kyaw Htet Aung, Hanley Kingston, Loice W. Mbogo, Betsy Sambai, Aliza Monroe-Wise, Natasha T. Ludwig-Barron, David Bukusi, William Sinkele, Esther Gitau, Sarah Masyuko, Joshua T. Herbeck, Carey Farquhar, Brandon L. Guthrie

**Affiliations:** 1 University of Washington, Seattle, WA USA; 2 University of Washington Global Assistance Program-Kenya, Nairobi, Kenya; 3 Kenyatta National Hospital, Nairobi, Kenya; 4 Support for Addiction Prevention and Treatment in Africa (SAPTA), Nairobi, Kenya; 5 National AIDS and STI Control Programme (NASCOP), Kenya Ministry of Health, Nairobi, Kenya

**Keywords:** Violence, HIV, Drug Use, People who inject drugs, Kenya

## Abstract

**Background:**

In Kenya, violence is common among people who inject drugs (PWID) living with HIV and their sexual and injecting partners and may lead to decreased uptake of HIV services, increased HIV risk behaviors, and increased HIV transmission. Violence is defined as any physical harm, threatened harm, or forced sexual acts inflicted on a person in the past year. Understanding the nature of violence and its correlates among PWID and their partners will inform population-specific public health interventions and policy recommendations.

**Methods:**

This is a cross-sectional study nested in a prospective cohort study conducted in eight public health centers, methadone clinics, and needle syringe programs in Nairobi, Kilifi, and Mombasa counties in Kenya. 3,302 sexual and/or injecting partners of PWID living with HIV were recruited through assisted partner services and participated in the study. Prevalence and correlates of violence were identified using the Wald test and negative binomial regression.

**Results:**

Out of 3302 study participants, 1439 (44%) had experienced violence within the past year. Physical violence was the most common form of violence experienced (35%), followed by being threatened (23%) or subjected to sexual violence (7%). In an adjusted analysis, female participants reported higher experiences of sexual violence (prevalence ratio [PR] = 2.46; 95% confidence interval [CI] 1.62, 3.74; *p* < 0.001) compared to male participants. In adjusted analysis, coastal residents had a higher experience of overall violence (PR = 1.48; 95% CI 1.27, 1.72; *p* < 0.001) than those living in Nairobi. This regional effect was relatively stronger among the female respondents (*p*_interaction_ = 0.025). Participants’ sex modified the association between region and experiencing violence after adjusting potential confounding factors.

**Conclusions:**

The study reveals the prevalence of violence among PWID and identifies high-risk sub-groups, including women, specifically for sexual violence, and coastal residents. Tailored interventions addressing their unique needs are essential. A holistic approach that combines violence prevention and response, comprehensive harm reduction, healthcare access, and community support is crucial to address the complex issue of drug use and HIV burden among PWID in Kenya for improved health outcomes.

## Introduction

With the joint third-largest HIV epidemic in the world, alongside Mozambique and Uganda, 1.5 million people were living with HIV in Kenya in 2019 [1, 2]. Kenya’s Ministry of Health has identified key populations that the HIV epidemic disproportionately impacts, including people who inject drugs (PWID), sex workers, and men who have sex with men (MSM). According to 2020 data from the Joint United Nations Programme on HIV/AIDS (UNAIDS), an estimated 18.3% of PWID were living with HIV in Kenya compared to 4.2% (3.7–4.9%) among the general adult population aged 15 to 49 years [1, 3].

Violence is also a leading public health problem globally, especially among people aged 15–44 years. According to the 2014 global status report on violence prevention, 2.5% of global mortality (1.3 million deaths) is a result of violence [4]. In Kenya, violence against PWID is commonly perpetrated by police, law enforcers, other authority figures, community members, and domestic partners [5]. As the number of PWID increases in Kenya, reports of violence against PWID have also been increasing, from 121 cases in 2013 to 873 cases in 2017 [6]. As per the third national behavioral assessment survey among Kenyan key populations (KPs), 44% of PWID experienced violence [7] associated with arbitrary police sweeps, harassment, beatings, bribery, remand, and imprisonment in the past 6 months [8]. The existing Kenyan legal system criminalizes PWID and makes them more vulnerable to violence [9]. Therefore, the combined effect of these restrictive laws and policies, cultural norms, social stigma, and discrimination can increase the risk of physical and threatened violence among PWID and their partners and creates barriers to engaging in HIV-related health services [10].

Many studies report an association between violence and HIV infection among KPs [8, 11]. Violence against KPs results in a decrease in HIV services uptake and an increase in HIV risk, including unsafe sex and injecting practices (e.g., having sex without a condom and injecting drugs with used needles and syringes) [8, 12, 13]. Fear of violence, social stigma, and discrimination may result in prioritizing safety over concerns about HIV and service uptake. As per the UNAIDS data, Kenya achieved the 90-90-90 target for 2020 in two areas – achieving 90% of people living with HIV know their HIV status (first 90) and 92% of people on ART with suppressed viral load (third 90). However, only 82% of the people who knew their HIV status were on ART treatment, which highlights the gaps in accessing regular treatment and care services [14]. The combined epidemic of drug use, HIV, and violence among the underserved PWID population in Kenya makes it especially difficult to access healthcare, and thus they may contribute disproportionately to failure to reach the second 90 [15].

To address the HIV epidemic among PWID, it is not enough to focus only on HIV and drug use. It is important to understand how these health consequences are correlated with violence and underlying social and societal determinants. The national government needs to address the violence and its related factors for the safety and well-being of KPs and to achieve the 2025 95–95-95 target of Kenya's HIV prevention program [6]. In this secondary data analysis, we aimed to assess the prevalence and correlates of violence among the partners of PWID living with HIV in Kenya.

## Methods

### Study design and setting

This is a cross-sectional study nested in a prospective cohort study conducted in eight public health centers, needle syringe programs (NSP), and methadone clinics in Nairobi, Kilifi, and Mombasa counties in Kenya [16]. The primary cohort study was conducted from February 2018 to December 2021 and examined the effectiveness of peer-mediated assisted partner services (APS) in identifying, testing, and linking to care the partners of PWID living with HIV in Kenya. Assessing the risk of violence was part of the primary study.

### Study population

A total of 3,302 sexual and injecting partners of PWIDs living with HIV, recruited through APS, participated in this study. Partners were eligible to participate if they were ≥ 18 years of age at the time of enrollment, had sexual intercourse and/or injecting experiences in the past three years with an index PWID enrolled in the primary study, and provided written informed consent to participate in the study [16].

### Definition of violence

Violence was defined as any physical, threatened, or sexual harm inflicted on a person in the past year (a year prior to their enrollment in the study). Physical violence included being hit, slapped, kicked, or otherwise physically hurt. Threatened violence included being threatened with a weapon. Sexual violence included someone being forced to perform sexual acts against his/ her will by anyone. Potential perpetrators include spouses, family members, sexual partners, police officers, drug dealers, gang members, communities, and others [16].

### Data collection

Partners were contacted confidentially by trained peer educators without revealing the identity of index participants. They were invited to test for HIV and hepatitis C and asked if they would like to participate in the study. After conducting written informed consent, researchers collected information about socio-demographic characteristics, history of experiencing violence and instability, HIV, and hepatitis C virus (HCV) seropositivity, and drug use history of participants by using a structured questionnaire through open data kit (ODK) software on tablet devices. All collected data were encrypted and uploaded on the Kenya National AIDS and STI Control Programme (NASCOP) servers. All participants were compensated for their transportation expenses [16].

### Data analysis and statistical method

We selected 17 variables based on the research interest, hypotheses, and conceptual framework. The independent (exposure) variables included age, sex, marital status, income sources, housing, region, partner type, HIV status, ART status, drug injecting status, methadone treatment, and sex or gender of sexual partners. The dependent (outcome) variable was experiencing violence among the study participants (any physical, threatened, and/or sexual violence).

We performed Wald test to identify the correlates of different types of violence in the total sample and stratified by sex. We reported the proportions of violence in each group, prevalence ratio (PR) with 95% confidence intervals (CI), and *p* values. For multiple categorical variables (e.g., age groups, marital status, and types of employment), we did pairwise comparison. For variables significant in the bivariate analysis, we did an adjusted analysis using negative binomial regression. Potential confounders were selected a priori based on a conceptual framework specific to each association. As a secondary analysis, we used negative binomial regression to test whether sex modified the association between region and violence. The data analysis was done by using R statistical software (Version 4.1.1).

## Results

### Overall characteristics of the study population (Table 1)

**Table 1:** Socio-demographic characteristics of study participants stratified by Sex

Socio–demographic Characteristics		Total *N* = 3302	Male *N* = 2336 (71%)	Female *N* = 966 (29%)
*Age years (Median, IQR)*				
		33 (27–39)	34 (28–40)	30 (25–36)
*Region*				
	Nairobi	1714 (52%)	1088 (47%)	626 (65%)
	Coast	1588 (48%)	1248 (53%)	340 (35%)
*Marital status*				
	Single	1368 (41%)	898 (38%)	470 (49%)
	Married	857 (26%)	669 (29%)	188 (19%)
	Partnered	221 (7%)	114 (5%)	107 (11%)
	Divorced/ Separated/ Widowed	854 (26%)	654 (28%)	200 (21%)
	Missing data	2		
*Employment*				
	Formal	176 (5%)	133 (6%)	43 (4%)
	Self–employ	391 (12%)	252 (11%)	139 (14%)
	Informal	511 (15%)	465 (20%)	46 (5%)
	Illegal	491 (15%)	167 (7%)	324 (34%)
	No income or partner family support	256 (8%)	76 (3%)	180 (19%)
	Others unspecified and missing data	1477 (45%)		
*Housing*				
	Stable	2888 (87%)	2027 (87%)	861 (89%)
	Unstable	410 (12%)	307 (13%)	103 (11%)
	Missing data	4		
*Partner types*				
	Sexual	590 (18%)	367 (16%)	223 (23%)
	Injecting	2326 (70%)	1685 (72%)	641 (66%)
	Both injecting and sexual	380 (12%)	279 (12%)	101 (10%)
	Missing data	6		
*HIV status*				
	Negative	2697 (82%)	2046 (88%)	651 (67%)
	Positive	594 (18%)	280 (12%)	314 (33%)
	Missing data	11		
*ART status*				
	On ART	461 (14%)	221 (9%)	240 (25%)
	Not on ART	133 (4%)	59 (3%)	74 (8%)
	Not living with HIV	2708 (82%)		
*Injection drug use status*				
	Non–active	126 (4%)	74 (3%)	52 (5%)
	Active (injected ≥ 1time last month)	2622 (79%)	1909 (82%)	713 (74%)
	Incomplete and missing data	554 (17%)		
*Methadone treatment status*				
	Taking Methadone	676 (20%)	530 (23%)	146 (15%)
	Not taking Methadone	2289 (69%)	1633 (70%)	656 (68%)
	Never injected drugs	337 (10%)		
*Male participants’ sexual partners*				
	Only women	2138 (65%)	2138 (91.6%)	–
	Men (or both men and women)	190 (5.8%)	190 (8.1%)	–
	NA	8 (0.2%)	8 (0.3%)	–
*Female participants’ sexual partners*				
	Only men	892 (27%)	–	892 (92.3%)
	Women (or both men and women)	72 (2.2%)	–	72 (7.5%)
	NA	2 (0.06%)	–	2 (0.2%)

Among 3,302 participants, 71% were males (*n* = 2,336), with a median age of 34 years (interquartile range (IQR): 28, 40). Fifty-two percent (*n* = 1,714) of participants were recruited from the Nairobi region, while the remaining 48% (*n* = 1,558) were from Coastal Kenya. Overall, 70% (*n* = 2,326) of participants were identified as injecting partners of the index PWID, 18% (*n* = 590) were sexual partners, and 12% (*n* = 380) were both injecting and sexual partners. At enrollment, 41% (n = 1,368) of participants were single, 26% (*n* = 857) were married, 7% (*n* = 221) were partnered, and 26% (*n* = 854) were divorced, separated, or widowed. Most participants (87%, *n* = 2,888) had stable housing.

Eighteen percent (*n* = 594) of participants tested positive for HIV, and 78% (*n* = 461) of those were on ART. Most participants (79%, *n* = 2,622), reported active injection drug use (injected drugs ≥ 1 time in the past month), and an additional 4% (*n* = 126) had a history of injection drug use but were not currently actively injecting drugs. Only 20% (*n* = 676) of all participants were currently participating in a methadone program.

Among all the male participants, 92% (*n* = 2,138) reported having sex with women only (MSW), while the remaining 8% (*n* = 190) reported having sex with men (MSM; this group includes 26 men who have sex with men only, and 164 men who have sex with both men and women). Among MSM, 79% (*n* = 150) lived in coastal Kenya, and 21% (*n* = 40) lived in Nairobi. Among female participants, 92% (*n* = 892) reported having sex with men only (WSM), while 7% (*n* = 72) reported having sex with women (WSW; this group includes 10 women who have sex with women only and 62 women who have sex with both men and women). Among WSW, 47% (*n* = 34) were from coastal Kenya, and 53% (*n* = 38) lived in Nairobi (Tables 2, 3, 4, 5).

**Table 2:** Correlates of experiencing violence among all participants in the past year

Variables	Any Violence		Physical Violence		Threatened Violence		Sexual Violence	
	*n*/*N* (%)	PR[95% CI]	*n*/*N* (%)	PR[95% CI]	*n*/*N* (%)	PR[95% CI]	*n*/*N* (%)	PR[95% CI]
*Total*								
	1439/3302[44%]		1152/3302[35%]		758/3302[23%]		232/3302[7%]	
*Sex*								
Male	1074/2336[46%]	1(Ref)	839/2336 [36%]	1(Ref)	609/2336 [26%]	1(Ref)	124/2336 [5%]	1(Ref)
Female	365/966 [38%]	0.82 **[0.75, 0.9]	313/966 [32%]	0.9[0.81, 1]	149/966 [15%]	0.59 **[0.5, 0.7]	108/966 [11%]	2.11 **[1.65, 2.7]
*Age*								
18–30 years	542/1273 [43%]	1(Ref)	442/1273 [35%]	1(Ref)	273/1273 [21%]	1(Ref)	97/1273 [8%]	1(Ref)
31–40 years	623/1354 [46%]	1.08[0.99, 1.18]	504/1354 [37%]	1.07[0.97, 1.19]	333/1354 [25%]	1.15[1, 1.32]	105/1354 [8%]	1.02[0.78, 1.33]
> 40 years	274/675 [41%]	0.95[0.85, 1.07]	206/675 [31%]	0.88[0.77, 1.01]	152/675 [23%]	1.05[0.88, 1.25]	30/675 [4%]	0.58 *[0.39, 0.87]
*Region*								
Nairobi	595/1714 [35%]	1(Ref)	510/1714 [30%]	1(Ref)	260/1714 [15%]	1(Ref)	54/1714 [3%]	1(Ref)
Coast	844/1588 [53%]	1.53 **[1.41, 1.66]	642/1588 [40%]	1.36 **[1.24, 1.49]	498/1588 [31%]	2.07 **[1.81, 2.36]	178/1588 [11%]	3.56 **[2.64, 4.79]
*Marital Status*								
Single	537/1368 [39%]	1(Ref)	445/1368 [33%]	1(Ref)	265/1368 [19%]	1(Ref)	84/1368 [6%]	1(Ref)
Married	365/857 [43%]	1.08[0.98, 1.2]	276/857 [32%]	0.99[0.87, 1.12]	204/857 [24%]	1.23 *[1.05, 1.44]	71/857 [8%]	1.35[1, 1.83]
Partnered	121/221 [55%]	1.39 **[1.22, 1.6]	95/221 [43%]	1.32 *[1.11, 1.57]	72/221 [33%]	1.68 **[1.35, 2.09]	24/221 [11%]	1.77 *[1.15, 2.72]
Divorced/ Separated/ Widow	416/854 [49%]	1.24 **[1.13, 1.37]	336/854 [39%]	1.21 *[1.08, 1.35]	217/854 [25%]	1.31 **[1.12, 1.54]	53/854[6%]	1.01[0.72, 1.41]
*Employment*								
Formal	76/176 [43%]	1(Ref)	57/176 [32%]	1(Ref)	42/176 [24%]	1(Ref)	13/176 [7%]	1(Ref)
Self-employ	151/391 [39%]	0.89[0.72, 1.1]	119/391 [30%]	0.94[0.72, 1.22]	75/391 [19%]	0.8[0.58, 1.12]	26/391 [7%]	0.9[0.47, 1.71]
Informal	218/511 [43%]	0.99[0.81, 1.2]	172/511 [34%]	1.04[0.81, 1.33]	124/511 [24%]	1.02[0.75, 1.38]	16/511 [3%]	0.42 *[0.21, 0.86]
Illegal	243/491 [49%]	1.15[0.95, 1.39]	200/491 [41%]	1.26[0.99, 1.6]	121/491 [25%]	1.03[0.76, 1.4]	59/491 [12%]	1.63[0.92, 2.89]
No income or partner family support	95/256 [37%]	0.86[0.68, 1.08]	78/256 [30%]	0.94[0.71, 1.25]	50/256 [20%]	0.82[0.57, 1.18]	26/256 [10%]	1.38[0.73, 2.6]
*Housing*								
Stable	1238/2888 [43%]	1(Ref)	976/2888 [34%]	1(Ref)	646/2888 [22%]	1(Ref)	218/2888 [8%]	1(Ref)
Unstable	201/410 [49%]	1.14 *[1.03, 1.27]	176/410 [43%]	1.27 **[1.12, 1.44]	112/410 [27%]	1.22 *[1.03, 1.45]	14/410 [3%]	0.45 *[0.27, 0.77]
*Partner type*								
Injecting	988/2326 [42%]	1(Ref)	782/2326 [34%]	1(Ref)	512/2326 [22%]	1(Ref)	144/2326 [6%]	1(Ref)
Sexual	256/590 [43%]	1.02 [0.92, 1.13]	206/590 [35%]	1.04[0.92, 1.18]	154/590 [26%]	1.19 *[1.01, 1.39]	59/590 [10%]	1.62 *[1.21, 2.16]
Both sex and inject	191/380 [50%]	1.18 *[1.06, 1.32]	162/380 [43%]	1.27 **[1.11, 1.44]	90/380 [24%]	1.08[0.88, 1.31]	27/380 [7%]	1.15[0.77, 1.71]
*HIV status*								
Negative	1178/2697 [44%]	1(Ref)	937/2697 [35%]	1(Ref)	635/2697 [24%]	1(Ref)	177/2697 [7%]	1(Ref)
Positive	255/594 [43%]	0.98[0.89, 1.09]	211/594 [36%]	1.02[0.91, 1.15]	120/594 [20%]	0.86[0.72, 1.02]	54/594 [9%]	1.39 *[1.03, 1.85]
*ART*								
On ART	199/461 [43%]	1(Ref)	163/461 [35%]	1(Ref)	90/461 [20%]	1(Ref)	35/461 [8%]	1(Ref)
Not on ART	56/133 [42%]	0.98[0.78, 1.22]	48/133 [36%]	1.02[0.79, 1.32]	30/133 [23%]	1.16[0.8, 1.67]	19/133 [14%]	1.88 *[1.11, 3.18]
*Injecting drug use and Methadone status*								
No active IDU/Methadone	19/69 [28%]	1(Ref)	18/69 [26%]	1(Ref)	7/69 [10%]	1(Ref)	1/69 [1%]	1(Ref)
Active IDU/Methadone	229/475 [48%]	1.75 *[1.18, 2.6]	182/475 [38%]	1.47 [0.97, 2.22]	123/475 [26%]	2.55 *[1.24, 5.24]	56/475 [12%]	8.13 *[1.14, 57.82]
Active IDU/ No Methadone	904/2147 [42%]	1.53 *[1.04, 2.25]	729/2147 [34%]	1.3 [0.87, 1.94]	445/2147 [21%]	2.04 *[1.01, 4.14]	104/2147 [5%]	3.34 [0.47, 23.6]
No active IDU/ No Methadone	26/57 [46%]	1.66 *[1.03, 2.67]	22/57 [39%]	1.48 [0.88, 2.48]	12/57 [21%]	2.08 [0.87, 4.92]	5/57 [9%]	6.05 [0.73, 50.33]

^*^Significant at 0.05 alpha level (*p* value < 0.05)

^**^Significant at 0.001 alpha level (*p* value < 0.001)

**Table 3:** Correlates of experiencing violence among male participants in the past year

Variables	Any Violence		Physical Violence		Threaten Violence		Sexual Violence	
	*n*/*N* (%)	PR[95% CI]	*n*/*N* (%)	PR[95% CI]	*n*/*N* (%)	PR[95% CI]	*n/N* (%)	PR[95% CI]
*Total*								
	1074/2336[46%]		839/2336[36%]		609/2336[26%]		124/2336[5%]	
*Age*								
18–30 years	362/775 [47%]	1(Ref)	287/775 [37%]	1(Ref)	205/775 [26%]	1(Ref)	42/775 [5%]	1(Ref)
31–40 years	477/999 [48%]	1.02 [0.93, 1.13]	379/999 [38%]	1.02 [0.91, 1.16]	268/999 [27%]	1.01 [0.87, 1.19]	66/999 [7%]	1.22 [0.84, 1.77]
> 40 years	235/562 [42%]	0.9 [0.79, 1.01]	173/562 [31%]	0.83 *[0.71, 0.97]	136/562 [24%]	0.91 [0.76, 1.1]	16/562 [3%]	0.53 *[0.3, 0.92]
*Region*								
Nairobi	422/1088 [39%]	1(Ref)	366/1088 [34%]	1(Ref)	213/1088 [20%]	1(Ref)	16/1088 [1%]	1(Ref)
Coast	652/1248 [52%]	1.35 **[1.23, 1.48]	473/1248 [38%]	1.13 *[1.01, 1.26]	396/1248 [32%]	1.62 **[1.4, 1.87]	108/1248 [9%]	5.88 **[3.5, 9.89]
*Marital Status*								
Single	383/898 [43%]	1(Ref)	316/898 [35%]	1(Ref)	202/898 [22%]	1(Ref)	43/898 [5%]	1(Ref)
Married	298/669 [45%]	1.04 [0.93, 1.17]	218/669 [33%]	0.93 [0.8, 1.07]	174/669 [26%]	1.16 [0.97, 1.38]	50/669 [7%]	1.56 * [1.05, 2.32]
Partnered	66/114 [58%]	1.36 *[1.14, 1.62]	47/114 [41%]	1.17 [0.92, 1.48]	47/114 [41%]	1.83 ** [1.43, 2.35]	9/114 [8%]	1.65 [0.83, 3.29]
Divorced/Separated/Widow	327/654 [50%]	1.17 *[1.05, 1.31]	258/654 [39%]	1.12 [0.98, 1.28]	186/654 [28%]	1.26 *[1.06, 1.5]	22/654 [3%]	0.7 [0.42, 1.16]
*Employment*								
Formal	54/133 [41%]	1(Ref)	38/133 [29%]	1(Ref)	28/133 [21%]	1(Ref)	7/133 [5%]	1(Ref)
Self-employ	102/252 [40%]	1 [0.77, 1.29]	75/252 [30%]	1.04 [0.75, 1.45]	57/252 [23%]	1.07 [0.72, 1.6]	10/252 [4%]	0.75 [0.29, 1.94]
Informal	204/465 [44%]	1.08 [0.86, 1.36]	160/465 [34%]	1.2 [0.9, 1.62]	117/465 [25%]	1.2 [0.83, 1.72]	15/465 [3%]	0.61 [0.26, 1.47]
Illegal	94/167 [56%]	1.39 *[1.08, 1.77]	74/167 [44%]	1.55 *[1.13, 2.13]	63/167 [38%]	1.79 * [1.22, 2.63]	10/167 [6%]	1.14 [0.45, 2.91]
No income or partner family support	27/76 [36%]	0.88 [0.61, 1.26]	21/76 [28%]	0.97 [0.62, 1.52]	18/76 [24%]	1.12 [0.67, 1.89]	5/76 [7%]	1.25 [0.41, 3.8]
*Housing*								
Stable	916/2027 [45%]	1(Ref)	700/2027 [35%]	1(Ref)	518/2027 [26%]	1(Ref)	119/2027 [6%]	1(Ref)
Unstable	158/307 [51%]	1.14 *[1.01, 1.28]	139/307 [45%]	1.31 **[1.14, 1.5]	91/307 [30%]	1.16 [0.96, 1.4]	5/307 [2%]	0.28 **[0.11, 0.67]
*Partner type*								
Injecting	772/1685 [46%]	1(Ref)	599/1685 [36%]	1(Ref)	438/1685 [26%]	1(Ref)	82/1685 [5%]	1(Ref)
Sexual	169/367 [46%]	1.01[0.89, 1.14]	132/367 [36%]	1.01[0.87, 1.18]	104/367 [28%]	1.09[0.91, 1.31]	26/367 [7%]	1.46[0.95, 2.23]
Both sex and inject	130/279 [47%]	1.02[0.89, 1.17]	107/279 [38%]	1.08[0.92, 1.27]	66/279 [24%]	0.91[0.73, 1.14]	15/279 [5%]	1.1[0.65, 1.89]
*HIV status*								
Negative	938/2046 [46%]	1(Ref)	739/2046 [36%]	1(Ref)	529/2046 [26%]	1(Ref)	107/2046 [5%]	1(Ref)
Positive	130/280 [46%]	1.01 [0.89, 1.16]	96/280 [34%]	0.95 [0.8, 1.13]	77/280 [28%]	1.06 [0.87, 1.3]	16/280 [6%]	1.09 [0.66, 1.82]
*ART*								
On ART	103/221 [47%]	1(Ref)	76/221 [34%]	1(Ref)	59/221 [27%]	1(Ref)	8/221 [4%]	1(Ref)
Not on ART	27/59 [46%]	0.98 [0.72, 1.34]	20/59 [34%]	0.99 [0.66, 1.47]	18/59 [31%]	1.14 [0.73, 1.78]	8/59 [14%]	3.75 *[1.47, 9.56]
*Injecting drug use and Methadone status*								
No active IDU/Methadone	13/47 [28%]	1(Ref)	12/47 [26%]	1(Ref)	6/47 [13%]	1(Ref)	0/47 [0%]	1(Ref)
Active IDU/Methadone	172/367 [47%]	1.69 *[1.05, 2.72]	134/367 [37%]	1.43 [0.86, 2.37]	101/367 [28%]	2.16 *[1.0, 4.64]	36/367 [10%]	Inf *[NaN, Inf]
Active IDU/ No Methadone	696/1542 [45%]	1.63 *[1.02, 2.6]	554/1542 [36%]	1.41 [0.86, 2.3]	371/1542 [24%]	1.88 [0.89, 4]	50/1542 [3%]	Inf [NaN, Inf]
No active IDU/ No Methadone	13/27 [48%]	1.74 [0.95, 3.19]	10/27 [37%]	1.45 [0.73, 2.9]	8/27 [30%]	2.32 [0.9, 5.98]	2/27 [7%]	Inf [NaN, Inf]
*Male participants’ sexual partners*								
Only women	957/2138 [45%]	1(Ref)	751/2138 [35%]	1(Ref)	518/2138 [24%]	1(Ref)	93/2138 [4%]	1(Ref)
Men (or both men and women)	116/190 [61%]	1.36 **[1.21, 1.54]	87/190 [46%]	1.3 *[1.11, 1.54]	90/190 [47%]	1.96 **[1.65, 2.31]	31/190 [16%]	3.75 **[2.57, 5.48]

^*^Significant at 0.05 alpha level (*p* value < 0.05)

^**^Significant at 0.001 alpha level (*p* value < 0.001)

**Table 4:** Correlates of experiencing violence among female participants in the past year

Variables	Any Violence		Physical Violence		Threaten Violence		Sexual Violence	
	*n*/*N* (%)	PR[95% CI]	*n*/*N* (%)	PR[95% CI]	*n*/*N* (%)	PR[95% CI]	*n*/*N* (%)	PR[95% CI]
*Total*								
	365/966[38%]		313/966[32%]		149/966[15%]		108/966[11%]	
*Age*								
18–30 years	180/498 [36%]	1(Ref)	155/498 [31%]	1(Ref)	68/498 [14%]	1(Ref)	55/498 [11%]	1(Ref)
31–40 years	146/355 [41%]	1.14 [0.96, 1.35]	125/355 [35%]	1.13 [0.93, 1.37]	65/355 [18%]	1.34 [0.98, 1.83]	39/355 [11%]	0.99 [0.68, 1.46]
> 40 years	39/113 [35%]	0.95 [0.72, 1.26]	33/113 [29%]	0.94 [0.68, 1.29]	16/113 [14%]	1.04 [0.63, 1.72]	14/113 [12%]	1.12 [0.65, 1.94]
*Region*								
Nairobi	173/626 [28%]	1(Ref)	144/626 [23%]	1(Ref)	47/626 [8%]	1(Ref)	38/626 [6%]	1(Ref)
Coast	192/340 [56%]	2.04 **[1.75, 2.39]	169/340 [50%]	2.16 **[1.81, 2.58]	102/340 [30%]	4 **[2.9, 5.5]	70/340 [21%]	3.39 **[2.34, 4.92]
*Marital Status*								
Single	154/470 [33%]	1(Ref)	129/470 [27%]	1(Ref)	63/470 [13%]	1(Ref)	41/470 [9%]	1(Ref)
Married	67/188 [36%]	1.09 [0.86, 1.37]	58/188 [31%]	1.12 [0.87, 1.46]	30/188 [16%]	1.19 [0.8, 1.78]	21/188 [11%]	1.28 [0.78, 2.11]
Partnered	55/107 [51%]	1.57 **[1.25, 1.96]	48/107 [45%]	1.63 **[1.26, 2.11]	25/107 [23%]	1.74 *[1.15, 2.63]	15/107 [14%]	1.61 [0.92, 2.79]
Divorced/Separated/Widow	89/200 [44%]	1.36 *[1.11, 1.66]	78/200 [39%]	1.42 *[1.13, 1.78]	31/200 [16%]	1.16 [0.78, 1.72]	31/200 [16%]	1.78 *[1.15, 2.75]
*Employment*								
Formal	22/43 [51%]	1(Ref)	19/43 [44%]	1(Ref)	14/43 [33%]	1(Ref)	6/43 [14%]	1(Ref)
Self-employ	49/139 [35%]	0.69 [0.48, 1]	44/139 [32%]	0.72 [0.47, 1.09]	18/139 [13%]	0.4 *[0.22, 0.73]	16/139 [12%]	0.82 [0.34, 1.98]
Informal	14/46 [30%]	0.59 [0.35, 1.01]	12/46 [26%]	0.59 [0.33, 1.07]	7/46 [15%]	0.47 [0.21, 1.05]	1/46 [2%]	0.16 [0.02, 1.24]
Illegal	149/324 [46%]	0.9 [0.66, 1.23]	126/324 [39%]	0.88 [0.61, 1.26]	58/324 [18%]	0.55 *[0.34, 0.9]	49/324 [15%]	1.08 [0.49, 2.38]
No income or partner family support	68/180 [38%]	0.74 [0.52, 1.04]	57/180 [32%]	0.72 [0.48, 1.07]	32/180 [18%]	0.55 *[0.32, 0.93]	21/180 [12%]	0.84 [0.36, 1.94]
*Housing*								
Stable	322/861 [37%]	1(Ref)	276/861 [32%]	1(Ref)	128/861 [15%]	1(Ref)	99/861 [11%]	1(Ref)
Unstable	43/103 [42%]	1.12 [0.87, 1.42]	37/103 [36%]	1.12 [0.85, 1.48]	21/103 [20%]	1.37 [0.91, 2.07]	9/103 [9%]	0.76 [0.4, 1.46]
*Partner type*								
Injecting	216/641 [34%]	1(Ref)	183/641 [29%]	1(Ref)	74/641 [12%]	1(Ref)	62/641 [10%]	1(Ref)
Sexual	87/223 [39%]	1.16[0.95, 1.41]	74/223 [33%]	1.16[0.93, 1.45]	50/223 [22%]	1.94 **[1.4, 2.69]	33/223 [15%]	1.53 *[1.03, 2.27]
Both sex and inject	61/101 [60%]	1.79 **[1.48, 2.17]	55/101 [54%]	1.91 **[1.54, 2.37]	24/101 [24%]	2.06 *[1.37, 3.1]	12/101 [12%]	1.23[0.69, 2.2]
*HIV status*								
Negative	240/651 [37%]	1(Ref)	198/651 [30%]	1(Ref)	106/651 [16%]	1(Ref)	70/651 [11%]	1(Ref)
Positive	125/314 [40%]	1.08 [0.91, 1.28]	115/314 [37%]	1.2 [1, 1.45]	43/314 [14%]	0.84 [0.61, 1.17]	38/314 [12%]	1.13 [0.78, 1.63]
*ART*								
On ART	96/240 [40%]	1(Ref)	87/240 [36%]	1(Ref)	31/240 [13%]	1(Ref)	27/240 [11%]	1(Ref)
Not on ART	29/74 [39%]	0.98 [0.71, 1.35]	28/74 [38%]	1.04 [0.75, 1.46]	12/74 [16%]	1.26 [0.68, 2.32]	11/74 [15%]	1.32 [0.69, 2.53]
*Injecting drug use and Methadone status*								
No active IDU/Methadone	6/22 [27%]	1(Ref)	6/22 [27%]	1(Ref)	1/22 [5%]	1(Ref)	1/22 [5%]	1(Ref)
Active IDU/Methadone	57/108 [53%]	1.94 *[0.96, 3.92]	48/108 [44%]	1.63 [0.8, 3.33]	22/108 [20%]	4.48 [0.64, 31.53]	20/108 [19%]	4.07 [0.58, 28.79]
Active IDU/ No Methadone	208/605 [34%]	1.26 [0.63, 2.52]	175/605 [29%]	1.06 [0.53, 2.12]	74/605 [12%]	2.69 [0.39, 18.48]	54/605 [9%]	1.96 [0.28, 13.55]
No active IDU/ No Methadone	13/30 [43%]	1.59 [0.72, 3.52]	12/30 [40%]	1.47 [0.65, 3.3]	4/30 [13%]	2.93 [0.35, 24.47]	3/30 [10%]	2.2 [0.24, 19.76]
*Female participants’ sexual partners*								
Only men	323/892 [36%]	1(Ref)	277/892 [31%]	1(Ref)	132/892 [15%]	1(Ref)	94/892 [11%]	1(Ref)
Women (or both men and women)	42/72 [58%]	1.61 **[1.3, 1.99]	36/72 [50%]	1.61 *[1.25, 2.07]	17/72 [24%]	1.6[1.02, 2.49]	14/72 [19%]	1.85 *[1.11, 3.07]

^*^Significant at 0.05 alpha level (*p* value < 0.05)

^**^Significant at 0.001 alpha level (*p* value < 0.001)

**Table 5:** Bivariate and multivariate analyses on the prevalence of overall violence among all participants in the past year

Predictor variables of interest	Any violence (Outcome variable)						
	Bivariate analysis			Multivariate analysis			Adjusted variables
	PR	95% CI	*p* value	PR	95% CI	*p* value	
*Sex*							
Male	1(Ref)						region, marital status, partner types, employment
Female	0.82	[0.75, 0.9]	< 0.001**	0.92	[0.77, 1.09]	0.31	
*Region*							
Nairobi	1(Ref)						sex, marital status, partner types, employment
Coast	1.53	[1.41—1.66]	< 0.001**	1.48	[1.27, 1.72]	< 0.001**	
*Region (Male only)*							
Nairobi	1(Ref)						marital status, partner types, employment
Coast	1.35	[1.23, 1.48]	< 0.001**	1.31	[1.08, 1.59]	0.007*	
*Region (Female only)*							
Nairobi	1(Ref)						marital status, partner types, employment
Coast	2.04	[1.75, 2.39]	< 0.001**	1.83	[1.42, 2.36]	< 0.001**	
*Marital Status*							
Single	1(Ref)						region, sex, partner types, employment
Married	1.08	[0.98, 1.2]	0.121	1.01	[0.83, 1.22]	0.957	
Partnered	1.39	[1.22, 1.6]	< 0.001**	1.26	[0.98, 1.62]	0.073	
Divorced/ Separated/ Widow	1.24	[1.13, 1.37]	< 0.001**	1.15	[0.96, 1.37]	0.143	
*Housing*							
Stable	1(Ref)						employment, sex, marital status, partner types
Unstable	1.14	[1.03, 1.27]	0.019*	1.07	[0.88, 1.32]	0.494	
*Injecting drug use and Methadone status*							
No active IDU/ Methadone	1(Ref)						marital status, partner types, employment
Active IDU/ Methadone	1.75*	[1.18, 2.6]	0.001*	1.50	[0.81, 2.79]	0.198	
Active IDU/ No Methadone	1.53*	[1.04, 2.25]	0.018*	1.39	[0.76, 2.54]	0.286	
No active IDU/ No Methadone	1.66*	[1.03, 2.67]	0.041*	1.55	[0.72, 3.36]	0.263	
*Male participants’ sexual partners*							
Only women	1(Ref)						region, marital status, partner types, employment
Men (or both men and women)	1.36	[1.21, 1.54]	< 0.001**	1.14	[0.84, 1.55]	0.397	
*Female participants’ sexual partners*							
Only men	1(Ref)						region, marital status, partner types, employment
Women (or both men and women)	1.61	[1.3, 1.99]	< 0.001**	1.27	[0.89, 1.81]	0.175	

^*^Significant at 0.05 alpha level (*p* value < 0.05)

^**^Significant at 0.001 alpha level (*p* value < 0.001)

### Correlates of violence among participants

Among all study participants, 44% had experienced any form of violence in the year prior to their enrollment. Physical violence was the most common form of violence reported (35%), followed by being threatened (23%) and sexual violence (7%). In bivariate analysis, experiencing overall violence was more common among males than females (PR = 1.22; 95%CI 1.11, 1.33; *p* < 0.001). After adjusting region, marital status, partner type, and employment, this association became insignificant (PR = 1.09; 95%CI 0.92, 1.29; *p* = 0.31). The prevalence of sexual violence was higher among female participants compared to males in both bivariate (PR = 2.11; 95%CI 1.65, 2.70; *p* < 0.001) and multivariate analyses (PR = 2.46; 95%CI 1.62, 3.74; *p* < 0.001) (Table 6).

**Table 6:** Bivariate and multivariate analyses on the prevalence of sexual violence among all participants in the past year

Predictor variables of interest	Sexual violence (outcome variable)						
	Bivariate analysis			Multivariate analysis			Adjusted variables
	PR	95% CI	*p* value	PR	95% CI	*p* value	
*Sex*							
Male	1(Ref)						region, marital status, partner types, employment
Female	2.11	[1.65, 2.7]	< 0.001**	2.46	[1.62, 3.74]	< 0.001**	
*Region*							
Nairobi	1(Ref)						sex, marital status, partner types, employment
Coast	3.56	[2.64, 4.79]	< 0.001**	3.06	[2.07, 4.52]	< 0.001**	
*Region (Male only)*							
Nairobi	1(Ref)						marital status, partner types, employment
Coast	5.88	[3.5, 9.89]	< 0.001**	3.65	[1.76, 7.56]	< 0.001**	
*Region (Female only)*							
Nairobi	1(Ref)						marital status, partner types, employment
Coast	3.39	[2.34, 4.92]	< 0.001**	3.15	[1.96, 5.07]	< 0.001**	
*Housing*							
Stable	1(Ref)						employment, sex, marital status, partner types, sex of sexual partners
Unstable	0.45	[0.27, 0.77]	0.001*	0.35	[0.16, 0.76]	0.008*	
*Housing (Male only)*							
Stable	1(Ref)						employment, marital status, partner types, sex of sexual partners
Unstable	0.28	[0.11, 0.67]	< 0.001**	0.09	[0.01, 0.74]	0.024*	
*Housing (Female only)*							
Stable	1(Ref)						employment, marital status, partner types, sex of sexual partners
Unstable	0.76	[0.4, 1.46]	0.509	0.54	[0.23, 1.27]	0.159	
*HIV status*							
Negative	1(Ref)						sex, region, marital status, partner types
Positive	1.39	[1.03, 1.85]	0.033*	0.89	[0.65, 1.24]	0.512	
*HIV status (Male only)*							
Negative	1(Ref)						region, marital status, partner types
Positive	1.09	[0.66, 1.82]	0.672	0.92	[0.54, 1.57]	0.762	
*HIV status (Female only)*							
Negative	1(Ref)						region, marital status, partner types
Positive	1.13	[0.78, 1.63]	0.586	0.89	[0.59, 1.34]	0.589	
*ART*							
On ART	1(Ref)						region, marital status, partner types, employment
Not on ART	1.88	[1.11, 3.18]	0.025*	1.79	[0.89, 3.63]	0.104	
*ART (Male only)*							
On ART	1(Ref)						region, marital status, partner types, employment
Not on ART	3.75	[1.47, 9.56]	0.008*	2.74	[0.53, 14.04]	0.227	
*ART (Female only)*							
On ART	1(Ref)						region, marital status, partner types, employment
Not on ART	1.32	[0.69, 2.53]	0.418	1.68	[0.73, 3.84]	0.219	

^*^Significant at 0.05 alpha level (*p* value < 0.05)

^**^Significant at 0.001 alpha level (*p* value < 0.001)

Participants living in coastal Kenya were more likely to have experienced any violence compared to those who lived in Nairobi in both bivariate (PR = 1.53; 95%CI 1.41, 1.66; *p* < 0.001), and multivariate analysis, adjusting for marital status, partner types, and employment (PR = 1.48; 95%CI 1.27, 1.72; *p* < 0.001) (Tables 2 and 5). This regional effect was stronger among the females (PR = 1.83; 95%CI 1.42, 2.36; *p* < 0.001) than the males (PR = 1.31; 95%CI 1.08, 1.59; *p* = 0.007) (*P*_interaction_ = 0.025) (Table 5). Participants’ sex modified the association between region and experiencing violence after adjusting potential confounding factors.

Compared to the participants who were single, those who had partners (PR = 1.39; 95%CI 1.22, 1.60; *p* < 0.001) or were divorced, separated, or widowed (PR = 1.24; 95%CI 1.13, 1.37; *p* < 0.001) reported higher experiences of violence in bivariate analysis (Table 2 and 5). However, this association became insignificant after adjusting for region, sex, partner types, and employment (Table 5).

Experiencing overall violence was more common among participants with no stable housing (PR = 1.14; 95%CI 1.03, 1.27; *p* = 0.019) in unadjusted analysis, but it was insignificant in adjusted analysis (PR = 1.07; 95%CI 0.88,1.32; *p* = 0.494) (Table 5). The association between unstable housing and experiencing sexual violence was significant in both bivariate (PR = 0.45; 95%CI 0.27, 0.77; *p* < 0.001) and multivariate analysis, adjusting for employment, sex, marital status, partner types, and sex of sexual partners (PR = 0.35; 95%CI 0.16,0.76; *p* = 0.008) (Table 6).

Furthermore, in bivariate analysis, violence was common among the participants those actively injecting drugs not taking methadone (PR = 1.53; 95% CI 1.04, 2.25, *p* = 0.018). Sexual partners of PWID were more likely than injecting partners of PWID to have experienced threatened (PR = 1.19; 95%CI 1.01, 1.39; *p* = 0.037) and sexual violence (PR = 1.62; 95%CI 1.21, 2.16; *p* = 0.002), while those who were both sexual and injecting partners reported higher experience of physical violence (PR = 1.27; 95%CI 1.11, 1.44; *p* < 0.001) (Table 2).

There was no evidence of a significant association between living with HIV or ART status and experiencing overall violence. However, in bivariate analysis, participants living with HIV were 1.39-times more likely (95%CI 1.03, 1.85; *p* = 0.033) to have experienced sexual violence than those without HIV. Among all the participants living with HIV, those not taking ART were 1.88-times more likely (95%CI 1.11, 3.18; *p* = 0.025) to have experienced sexual violence than those who were on ART (Table 2). The prevalence of experiencing sexual violence among males who did not take ART was 3.75-times higher (95%CI 1.47, 9.56; *p* = 0.008) than that of males who were on ART (Table 3). All these associations become insignificant after adjusting sex, region, marital status, partner type, and employment (Table 6).

In bivariate analysis, the sex of participants’ sexual partners was significantly associated with their likelihood of experiencing violence. MSM were 1.36-times more likely to have experienced violence (95%CI 1.21, 1.54; *p* < 0.001) compared to MSW, and similarly WSW were 1.61-times more likely to have experienced violence (95%CI 1.3, 1.99; *p* =  < 0.001) compared to WSM (Table 3 and 4). After adjusting for potential confounding factors including region, marital status, partner types, and employment, the association became insignificant (Table 5).

## Discussion

The study found a high prevalence of violence among sexual and injecting partners of people who inject drugs, with a rate of 44%. This finding is consistent with the 2018 NASCOP survey, which showed that 44% of PWID in Kenya experienced violence in the past 6 months [7]. In Kenya, people who use drugs are often convicted of theft due to their addiction being perceived as the reason for stealing. They face police brutality, bribery, and mob justice [17]. In 2021, mobs in Coastal Kenya killed six people and severely beat three others on suspicion of involvement in crime, with victims being burnt alive or stoned to death, and two suspected thieves beaten in Kilifi [18]. This has led to calls for law enforcement authorities to be trained to identify drug addiction as a health condition, as well as calls to intensify anti-stigma efforts and public awareness campaigns for community sensitization. Community-based initiatives could also be established to protect and provide support for people who use drugs and prevent mob violence.

The study underscored that participants who were actively injecting drugs were found to have relatively higher experiences of violence compared to non-active injecting drug users who were receiving methadone treatment, although it was not statistically significant in multivariate analysis. Active injecting drug users may face greater financial instability, which increases their vulnerability to violence from social and structural factors [19]. While it is challenging to establish causality from our findings, it is plausible that violence may contribute to disruptions in accessing regular methadone treatment among PWIDs. Prior studies have shown social and structural factors like violence, social stigma and discrimination, political and social inequalities, ineffective policies, laws, and policing, impair access to harm reduction services and are associated with a higher risk of HIV acquisition among PWID [20], while methadone maintenance treatment can reduce heroin use and subsequent crime and violence rates [21]. Therefore, creating safer environments, reducing structural barriers to healthcare access, and implementing effective violence prevention and response interventions are crucial to enhance PWIDs engagement in healthcare services like methadone treatment.

We also found that violence was more prevalent in Coastal Kenya than in Nairobi, with a greater regional effect for females than males, possibly due to differences in the cultural context and attitudes toward gender-based violence. According to a multidimensional analysis on poverty in Kenya, conducted in 2018, women in Coastal Kenya exhibited higher rates of low literacy and early marriage [22]. The 2018 Kenya NASCOP survey revealed that 12% of PWID had experienced forced sex in the last six months, with a higher prevalence among female PWID than male PWID [7]. According to a study conducted in Kenya, 80–100% of female drug users engaged in sex work to finance their own drug use as well as their partner’s [23]. Being female and using drugs can have a compounded effect, increasing vulnerability to power imbalances, difficulties in negotiating for safer sex, and a higher likelihood of being exposed to sexual violence [24]. Stigma related to drug use, gender, and HIV status can lead to exclusion, low self-esteem, and difficulty accessing healthcare services for women who inject drugs in Kenya [25]. Interventions that address cultural attitudes toward gender-based violence, improve literacy rates, and provide access to healthcare services for women who inject drugs are essential to reduce violence and improve the health and well-being of women in Coastal Kenya.

The results from bivariate analysis among males reveal a strong association between experiencing sexual violence and some characteristics including region, sex of their sexual partners, and ART status, although not significant in multivariate analysis. Previous studies reported higher rates of violence among MSM in the Coastal region [26] and among those engaged in sex work [27]. The combined effects of being MSM, engaging in drug use and sex work, criminalization, and discrimination may make this sub-population more vulnerable and stigmatized. Therefore, along with the other groups of PWID, including women, MSM who inject drugs, and male sex workers who inject drugs, may need special attention and targeted interventions. It is also essential to address underlying factors contributing to violence, such as gender inequality and stigma toward PWID and individuals living with HIV (Table 6).

Furthermore, we observed that the prevalence of experiencing sexual violence was higher within the stable housing group compared to those without stable housing, both in unadjusted and adjusted analyses. This finding is surprising and counter-intuitive in the context of real-world scenarios. Possible explanations for this unexpected association include the likelihood that individuals without stable housing may have fewer or no regular sexual partners, thus reducing opportunities for experiencing intimate partner violence (IPV). Additionally, reporting bias may be a factor, as individuals without stable housing may be less inclined to report incidents of sexual violence. Furthermore, it is possible that there are other significant confounding variables that we did not account for. Additional research is essential to gain a deeper understanding of the complex relationship between sexual violence and stable housing.

### Study limitations

The study has limitations. Our data on violence were limited because we only asked about violence in the last year and we excluded participants who experienced violence in the month prior to enrollment. Longitudinal data would help elucidate whether the associations we found were causal, and community-level data could further reveal complex contributions to risk of violence. The gender distribution in our study might not fully represent the broader population of people who inject drugs (PWID) in Kenya, where the usual distribution is predominantly male (around 82–89%) as reported in existing research [28]. This variation could be due to inclusion of sexual partners of PWID, which enriches the number of female participants in study sample. In the data collection process, we utilized the question 'What is your sex? (Male or Female)' to gather information related to gender. It is important to acknowledge that this question does not capture the complexities of sex and gender identity, as it does not distinguish between sex assigned at birth, current physiological sex, and gender identity. Moreover, we did not distinguish between sex and gender when asking about sexual partners. Nonetheless, the study's results provide valuable insight into the prevalence, distribution, and potential correlates of violence among PWID in Kenya. Future research could employ a socio-ecological model to examine violence across different levels and investigate effective interventions for this vulnerable population.

## Conclusions

In conclusion, this study confirms the high prevalence of violence among PWID and their partners and shows that specific sub-groups are at even higher risk, such as women specifically for sexual violence, and those living on the coast. The study underscores the need for interventions that address the underlying determinants and specific needs of these sub-groups. A holistic approach that combines violence prevention and response with comprehensive harm reduction interventions, healthcare access, and community support initiatives is essential to address the complex issue of drug use and HIV burden among PWID in Kenya and to achieve better health outcomes.

## Data Availability

The datasets used and/or analyzed during the current study are available from the corresponding author upon reasonable request.

## Abbreviations

APS: Assisted partner services
ART: Antiretroviral therapy
CI: Confidence intervals
HIV: Human immunodeficiency virus
KPs: Key populations
IDU: Injecting drug use
MSM: Men who have sex with men
MSMW: Men who have sex with both men and women
MSW: Men who have sex with women
NASCOP: National AIDS and STI control programme
ODK: Open data kit
PWID: People who inject drugs
PR: Prevalence ratio
UNAIDS: Joint United Nations programme on HIV/AIDS
UNOCHA: United Nations office for the coordination of humanitarian affairs
UNODC: United Nations office on drugs and crime
WHO: World health organization
WSM: Women who have sex with men
WSMW: Women who have sex with both men and women
WSW: Women who have sex with women
